# Impact of a local formulary on prescribing patterns and pharmaceutical expenditure: an observational study in Japan

**DOI:** 10.1186/s40780-025-00526-2

**Published:** 2025-12-22

**Authors:** Hirofumi Koike, Maho Taguchi, Akihiro Koide, Hiroaki Yamada

**Affiliations:** 1 Laboratory of Regulatory Science, Yokohama University of Pharmacy, 601 Matano-Cho, Totsuka-ku, Yokohama-shi, Kanagawa, 245-0006 Japan; 2 Department of Pharmacy, Yokohama City University Hospital, Yokohama, Japan

**Keywords:** Local formulary, Generic drugs, Medical costs, National Database, Japan

## Abstract

**Background:**

The implementation of local formularies in Japan began in November 2018 in the Sakata area of Yamagata Prefecture. A local formulary is a regional list of recommended pharmaceuticals selected based on comprehensive criteria, including efficacy, safety, and economic considerations. However, the economic impact of these formulations has not yet been clearly demonstrated. This retrospective observational study aimed to verify their effectiveness by analyzing real-world data.

**Methods:**

Prescription and outpatient data from the National Database were extracted for October 2018, 2019, and 2020, covering both the Sakata area and the rest of Yamagata Prefecture. The number of prescriptions and cost per prescription were calculated for four therapeutic classes: proton pump inhibitors/potassium-competitive acid blockers, angiotensin receptor blockers, α-glucosidase inhibitors, and statins.

**Results:**

In 2020, the formulary adherence rate in Sakata exceeded that in the rest of Yamagata Prefecture across all therapeutic classes. Although the cost per prescription annually decreased in Sakata, some therapeutic classes showed increased costs in the remainder of the prefecture from 2019 to 2020. In contrast, formulary adherence to outpatient medical prescriptions was lower in Sakata, and no clear impact of the local formulary was observed. Applying the rate of cost reduction observed in Sakata to the rest of the prefecture, the estimated monthly savings reached approximately 35 million JPY after 1 year and 15 million JPY after 2 years. Furthermore, although no prior measures existed to control the use of originator drugs without generics, Sakata showed a trend of reduced usage compared with the rest of the prefecture, suggesting the influence of the local formulary.

**Conclusions:**

Local formularies can contribute to the sustainability of Japan’s healthcare financing systems.

## Background

In Japan, various academic societies have developed clinical practice guidelines [1–4] to promote appropriate pharmaceutical use. However, the selection of recommended drugs in these guidelines primarily focuses on efficacy and safety, with a few examples incorporating economic considerations. Meanwhile, national healthcare expenditures continue to increase annually, and ensuring that prescriptions are made from both clinical and economic perspectives is an urgent issue for maintaining the universal health insurance system.

Formularies in Japan can be broadly categorized into two types: hospital formularies, implemented at the institutional level, and local formularies, developed collaboratively by regional stakeholders [5]. Both types primarily recommend generic drugs, and their introduction and expansion are expected to reduce pharmaceutical costs. Local formularies are defined as regional drug lists and usage policies that include medications deemed optimal from a comprehensive perspective, considering efficacy, safety, and cost, through collaboration among local physicians, pharmacists, and related organizations.

In contrast to formularies in some countries, such as the United States, which effectively restrict the use of certain drugs by insurers, concerns have been raised that Japan’s local formularies might similarly limit drug availability. However, the Japanese government has clarified that local formularies do not restrict drug use and that medications outside the formulary may be prescribed when deemed necessary [6].

A 2022 survey conducted by the Japan Society of Hospital Pharmacists revealed that 42.6% of the major hospitals in Japan had implemented hospital formularies [7]. Such implementation can reduce drug procurement costs [8–10]. However, because drug costs for inpatients are covered under the Diagnosis Procedure Combination system, financial benefits are limited to hospital management and do not directly impact national healthcare expenditures. Moreover, hospital formularies may influence outpatient prescriptions after discharge [11, 12], but their impact on overall national healthcare costs remains minimal owing to their limited scope.

In contrary, local formularies were first introduced in November 2018 in the Sakata area of Yamagata Prefecture (Sakata City, Yuza Town, Shonai Town) and have since been adopted in various regions across Japan. By consolidating therapeutically equivalent drugs into recommended options, local formularies are expected to reduce the burden of inventory management in pharmacies and improve the distribution efficiency among wholesalers. Additionally, increased prescription rates of cost-effective recommended drugs directly contribute to reduced pharmaceutical expenditures. However, to date, no study has used real-world data.

Japan’s pharmaceutical cost containment strategies include periodic drug price revisions, promotion of generic and biosimilar drug use, and efforts to reduce polypharmacy and duplicate prescriptions through appropriate use. However, there is no mention of restrictions on the use of originator drugs without available generics. Because local formularies do not recommend originator drugs, it is necessary to clarify their impact.

This study aimed to evaluate the economic impact of introducing a local formulary in the Sakata area by analyzing receipt data from the National Database of Health Insurance Claims and Specific Health Checkups of Japan **(**NDB) before and after 1 and 2 years after implementation. The rest of Yamagata Prefecture, excluding Sakata, was used as the comparison group.

Yamagata Prefecture, located in the Tohoku region of northern Japan, had an estimated population of approximately 1.06 million as of 2020. The Sakata area, situated in the northwestern part of the prefecture, including Sakata City, ranks as the third-largest municipality in Yamagata.

As of 2020, the healthcare infrastructure across Yamagata Prefecture consisted of 67 hospitals, 910 clinics, 813 clinic-based physicians, 602 pharmacies, and 1,359 pharmacists working in pharmacies. The Sakata area specifically contained 7 hospitals, 116 clinics, 103 clinic-based physicians, 55 pharmacies, and 144 pharmacists. These figures reflect the region’s commitment to maintaining accessible medical services, despite demographic challenges (such as population decline and aging).

## Methods

### Target drug classes for analysis

Among the six drug classes included in the local formulary that had been in operation for over 1 year in Sakata as of October 2020, four classes were selected for analysis: oral acid secretion inhibitors (proton pump inhibitors [PPIs]/potassium-competitive acid blockers [P-CABs]), angiotensin receptor blockers (ARBs), postprandial hyperglycemia-improving agents (α-GIs), and 3-hydroxy-3-methylglutaryl-CoA reductase inhibitors (statins). The formulary and non-formulary drugs in each class are listed in Table 1.

**Table 1:** The Sakata local formulary

Therapeutic class	Formulary drugs	Non-formulary drugs
PPI/P-CAB^a)^	Lansoprazole (generic)	Lansoprazole (originator)
	Omeprazole (generic)	Omeprazole (originator)
	Rabeprazole (generic)	Rabeprazole (originator)
		Esomeprazole^c)^
		Vonoprazan^c)^
ARB^b)^	Candesartan (generic)	Candesartan (originator)
	Olmesartan (generic)	Olmesartan (originator)
	Telmisartan (generic)	Telmisartan (originator)
		Azilsartan^c)^
		Irbesartan
		Losartan
		Valsartan
α-GI^a)^	Miglitol (generic)	Miglitol (originator)
	Voglibose (generic)	Voglibose (originator)
		Acarbose
Statin^b)^	Pitavastatin (generic)	Pitavastatin (originator)
	Rosuvastatin (generic)	Rosuvastatin (originator)
		Atorvastatin
		Fluvastatin
		Pravastatin
		Simvastatin

^a)^Introduced in November 2018

^b)^Introduced in February 2019

^c)^Originator drugs without currently available generics

The remaining two classes were excluded from the analysis: infliximab, as it is an injectable infusion drug not handled by community pharmacies, and oral bisphosphonates, because of the wide variation in dosing schedules (daily, weekly, and monthly), which makes quantitative comparison difficult.

For combination drugs, generics containing the same active ingredients as the recommended drugs were classified as formulary drugs, whereas originator drugs were classified as non-formulary drugs.

As no other local formulary exists in Yamagata Prefecture outside Sakata, this classification was applied consistently across the region.

### Survey items and study period

The analysis targeted prescription claims (receipts) for the selected drug classes from both community pharmacies and outpatient medical institutions in Sakata and the rest of Yamagata Prefecture.

The study period covered 3 months: October 2018, October 2019, and October 2020. For each drug, the numbers of prescriptions and pharmaceutical expenditures were calculated. Drug costs were computed using the official drug prices for each respective year.

The data source was the National Database of Health Insurance Claims and Specific Health Checkups of Japan (NDB), provided by the Ministry of Health, Labour and Welfare under approval number 1510, dated July 1, 2023.

Descriptive statistics were used to compare the number of prescriptions and pharmaceutical expenditures over time and between regions. No formal hypothesis testing or power calculations were performed, as the objective was to assess real-world trends rather than test a specific statistical hypothesis.

### Data cleansing

The following data were excluded from analysis:

Cases in which the daily dosage exceeded three times the maximum dose specified in the Japanese package insert, prescriptions with fewer than three units dispensed, and duplicate entries for the same prescription date.

### Ethical considerations

This study was conducted in accordance with the Ethical Guidelines for Life Science and Medical Research Involving Human Subjects and was approved by the Clinical Research Ethics Review Committee of Yokohama University of Pharmacy (Approval No.: C23011A, dated July 5, 2023).

## Results

The number of prescription claims analyzed based on drug class and region during the study period is presented in Table 2. The total number of claims increased from 356,466 in 2018 to 366,989 in 2020, representing an approximate 3.0% increase over 2 years. Based on drug class, the number of claims for PPIs/P-CABs increased by 8.5% and statins by 5.3%, whereas ARBs decreased by 0.5% and α-GIs by 12.0%. Of the total claims, 80.8% were from community pharmacies and 19.2% from medical institutions, indicating that pharmacy claims have a greater impact on pharmaceutical expenditures in regional health care.

**Table 2:** Results of the analyzed data

Category	Therapeutic class	PPI/*P*-CAB			ARB			α-GI			Statin			Total
	Survey year	2018	2019	2020	2018	2019	2020	2018	2019	2020	2018	2019	2020	-
Pharmacy	Yamagata Pref.^a)^	57,232	60,136	63,246	107,936	107,771	108,847	9,454	8,890	8,372	80,709	82,695	86,161	781,449
	Sakata area	7,491	8,285	8,888	10,714	11,072	11,845	913	850	895	9,742	10,352	11,610	92,657
Medical	Yamagata Pref.a)	9,402	8,989	9,064	24,544	23,145	22,874	1,921	1,716	1,626	20,452	20,257	20,239	164,229
	Sakata area	3,306	3,124	2,808	6,273	5,395	5,161	317	272	195	6,060	5,892	5,158	43,961
	Total	77,431	80,534	84,006	149,467	147,383	148,727	12,605	11,728	11,088	116,963	119,196	123,168	1,082,296

^a)^Excluding Sakata

**Table 3:** Claims analysis results (volume)

Therapeutic class	Study area	Category of drugs	Claims count			Formulary adherence rate			
			2018	2019	2020	2018	2019	2020	Growth rate (2018→2020)
**A Pharmacy**									
PPI/P-CAB	Yamagata Pref.	Formulary	794,290	859,961	980,561	45.6%	46.2%	47.9%	2.3%
		Non-formulary	949,446	1,002,568	1,067,204				
	Sakata area	Formulary	118,979	166,495	193,365	50.1%	62.8%	65.8%	15.7%
		Non-formulary	118,372	98,629	100,315				
ARB	Yamagata Pref.	Formulary	1,555,295	1,643,601	1,797,154	42.9%	44.7%	46.3%	3.4%
		Non-formulary	2,068,345	2,029,257	2,085,951				
	Sakata area	Formulary	155,857	191,995	232,711	42.4%	50.2%	54.1%	11.7%
		Non-formulary	212,022	190,456	197,741				
α-GI	Yamagata Pref.	Formulary	611,290	591,291	605,680	70.7%	72.8%	75.3%	4.7%
		Non-formulary	253,928	220,826	198,228				
	Sakata area	Formulary	72,118	67,833	72,179	82.8%	84.2%	86.0%	3.3%
		Non-formulary	15,019	12,757	11,706				
Statin	Yamagata Pref.	Formulary	1,129,119	1,246,683	1,440,212	41.1%	43.5%	46.1%	5.0%
		Non-formulary	1,620,871	1,616,872	1,687,064				
	Sakata area	Formulary	147,223	190,954	228,169	43.5%	52.7%	54.2%	10.7%
		Non-formulary	191,452	171,575	193,009				
**B Medical**									
PPI/P-CAB	Yamagata Pref.	Formulary	201,471	198,741	205,555	65.7%	67.0%	66.7%	0.9%
		Non-formulary	104,952	97,681	102,853				
	Sakata area	Formulary	55,715	60,195	53,720	52.2%	58.1%	56.4%	4.2%
		Non-formulary	51,011	43,396	41,568				
ARB	Yamagata Pref.	Formulary	373,486	411,719	402,770	43.3%	50.2%	47.9%	4.6%
		Non-formulary	488,301	408,059	437,376				
	Sakata area	Formulary	50,854	80,601	78,193	22.6%	40.9%	40.4%	17.8%
		Non-formulary	174,445	116,489	115,438				
α-GI	Yamagata Pref.	Formulary	118,171	110,930	108,632	65.5%	68.7%	69.8%	4.3%
		Non-formulary	62,197	50,561	47,048				
	Sakata area	Formulary	12,731	13,418	6,862	42.2%	49.0%	32.1%	-10.2%
		Non-formulary	17,409	13,991	14,525				
Statin	Yamagata Pref.	Formulary	204,848	234,038	262,528	28.8%	32.6%	35.3%	6.5%
		Non-formulary	507,173	483,448	481,418				
	Sakata area	Formulary	33,640	38,300	34,359	15.8%	18.3%	18.0%	2.3%
		Non-formulary	179,575	171,454	156,028				

**Table 4:** Claims analysis results (cost, JPY)

Therapeutic class	Study area	Category of drugs	Survey year			Percentage change					
			2018	2019	2020	2018→2019		2019→2020		2018→2020	
**A Pharmacy**											
PPI/P-CAB	Yamagata Pref.	Formulary	29,972,700	28,167,050	30,061,198	-6.0%	2.4%	6.7%	7.9%	0.3%	10.5%
		Non-formulary	109,333,832	114,469,012	123,819,081	4.7%		8.2%		13.2%	
	Sakata area	Formulary	4,289,341	5,121,999	5,694,155	19.4%	-6.6%	11.2%	4.4%	32.8%	-2.5%
		Non-formulary	13,013,831	11,040,133	11,173,035	-15.2%		1.2%		-14.1%	
	Subtotal		156,609,704	158,798,194	170,747,469		1.4%		7.5%		9.0%
ARB	Yamagata Pref.	Formulary	54,177,760	48,305,570	50,941,789	-10.8%	-8.9%	5.5%	1.3%	-6.0%	-7.7%
		Non-formulary	170,578,521	156,417,003	156,501,444	-8.3%		0.1%		-8.3%	
	Sakata area	Formulary	5,481,042	5,357,195	6,249,478	-2.3%	-12.1%	16.7%	4.0%	14.0%	-8.6%
		Non-formulary	18,748,039	15,936,439	15,900,020	-15.0%		-0.2%		-15.2%	
	Subtotal		248,985,363	226,016,207	229,592,730		-9.2%		1.6%		-7.8%
α-GI	Yamagata Pref.	Formulary	9,658,437	8,247,155	8,115,063	-14.6%	-14.8%	-1.6%	-6.5%	-16.0%	-20.3%
		Non-formulary	9,498,502	8,069,585	7,148,905	-15.0%		-11.4%		-24.7%	
	Sakata area	Formulary	1,125,109	933,888	968,972	-17.0%	-19.0%	3.8%	0.5%	-13.9%	-18.6%
		Non-formulary	629,742	488,395	459,808	-22.4%		-5.9%		-27.0%	
	Subtotal		20,911,790	17,739,024	16,692,748		-15.2%		-5.9%		-20.2%
Statin	Yamagata Pref.	Formulary	29,717,315	26,372,301	29,130,515	-11.3%	-10.2%	10.5%	4.0%	-2.0%	-6.6%
		Non-formulary	63,118,786	57,031,987	57,602,521	-9.6%		1.0%		-8.7%	
	Sakata area	Formulary	4,004,460	4,240,662	4,995,764	5.9%	-9.5%	17.8%	11.9%	24.8%	1.3%
		Non-formulary	6,741,045	5,487,396	5,889,574	-18.6%		7.3%		-12.6%	
	Subtotal		103,581,605	93,132,346	97,618,375		-10.1%		4.8%		-5.8%
Total			530,088,462	495,685,771	514,651,322		6.5%		3.8%		2.9%
**B Medical**											
PPI/P-CAB	Yamagata Pref.	Formulary	7,784,634	6,637,914	6,616,156	-14.7%	-9.9%	0.3%	4.0%	15.0%	-6.3%
		Non-formulary	11,930,358	11,118,771	11,851,809	-6.8%		6.6%		-0.7%	
	Sakata area	Formulary	2,038,205	1,926,245	1,513,118	-5.5%	-12.3%	-21.4%	-8.0%	-25.8%	-19.4%
		Non-formulary	5,754,105	4,906,260	4,770,757	-14.7%		-2.8%		-17.1%	
	Subtotal		27,507,301	24,589,191	24,751,838		-10.6%		0.7%		-10.0%
ARB	Yamagata Pref.	Formulary	12,466,431	13,530,153	10,685,306	8.5%	-14.8%	-21.0%	-4.9%	-14.3%	-19.0%
		Non-formulary	35,378,782	27,227,533	28,057,717	-23.0%		3.0%		-20.7%	
	Sakata area	Formulary	1,559,140	3,121,640	1,704,859	100.2%	-24.3%	-45.4%	-18.3%	9.3%	-38.2%
		Non-formulary	15,281,418	9,622,812	8,707,878	-37.0%		-9.5%		-43.0%	
	Subtotal		64,685,771	53,502,137	49,155,759		-17.3%		-8.1%		-24.0%
α-GI	Yamagata Pref.	Formulary	1,686,300	1,395,700	1,325,973	-17.2%	-19.6%	-5.0%	-8.1%	-21.4%	-26.1%
		Non-formulary	2,153,893	1,691,356	1,511,097	-21.5%		-10.7%		-29.8%	
	Sakata area	Formulary	179,342	163,937	82,399	-8.6%	-32.1%	-49.7%	-16.5%	-54.1%	-43.3%
		Non-formulary	528,721	316,696	318,937	-40.1%		0.7%		-39.7%	
	Subtotal		4,548,256	3,567,688	3,238,405		-21.6%		-9.2%		-28.8%
Statin	Yamagata Pref.	Formulary	5,253,967	4,771,832	5,200,450	-9.2%	-12.6%	9.0%	-2.5%	-1.0%	-14.7%
		Non-formulary	18,436,908	15,941,637	15,000,121	-13.5%		-5.9%		-18.6%	
	Sakata area	Formulary	825,761	785,646	692,625	-4.9%	-12.3%	-11.8%	-8.3%	-16.1%	-19.5%
		Non-formulary	7,668,970	6,666,173	6,142,433	-13.1%		-7.9%		-19.9%	
	Subtotal		32,185,607	28,165,289	27,035,628		-12.5%		-4.0%		-16.0%
Total			128,926,935	109,824,306	104,181,630		-14.8%		-5.1%		-19.2%

A comparison of prescription volumes in pharmacy claims between 2018 and 2020 revealed that in the PPI/P-CAB group, both formulary and non-formulary drug volumes increased in Yamagata Prefecture. In contrast, in Sakata, formulary drugs increased by 62.5%, whereas non-formulary drugs decreased by 15.3%. In the ARB group, formulary drugs increased by 15.6% and non-formulary drugs by 0.9% across the entire prefecture, whereas in Sakata, formulary drugs increased by 49.3% and non-formulary drugs decreased by 6.7%. In the α-GI group, formulary drug volumes remained relatively stable in both regions, whereas non-formulary drugs decreased by > 20%. In the statin group, formulary drugs increased by 27.6% and non-formulary drugs by 4.1% across the entire prefecture, whereas in Sakata, formulary drugs increased by 55.0% and non-formulary drugs increased by only 0.8% (Table 3A).

The formulary compliance rates increased over time in both regions. Notably, for PPI/P-CABs and ARBs, the increases in Yamagata Prefecture were 2.3% and 3.4%, respectively, whereas in Sakata, these rates increased significantly by 15.7% and 11.7%, respectively.

Regarding medical institution claims, the PPI/P-CAB group showed little change in the overall prefecture; however, in Sakata, non-formulary drugs decreased by 18.5%, resulting in a 4.2% increase in compliance. In the ARB group, formulary drugs increased by 7.8% and non-formulary drugs decreased by 10.4% in the prefecture, whereas in Sakata, formulary drugs increased by 53.8% and non-formulary drugs decreased by 33.8%, leading to a 17.8% increase in compliance. In the α-GI group, both regions saw a decrease in volume, but in Sakata, formulary drugs decreased by 46.1%, resulting in a 10.2% decline in compliance. In the statin group, formulary drugs increased by 28.2% and non-formulary drugs decreased by 5.1% in the prefecture, whereas in Sakata, formulary drugs increased by 2.1% and non-formulary drugs decreased by 13.1%, leading to a 2.3% increase in compliance. Although Sakata showed higher growth rates in compliance with PPIs/P-CABs and ARBs, the overall compliance rates in medical institution claims were lower than those in the rest of Yamagata Prefecture (Table 3B).

The pharmaceutical expenditures for pharmacy claims are listed in Table 4A. Total costs decreased by 6.5% from 2018 to 2019 but increased by 3.8% from 2019 to 2020. From 2018 to 2020, expenditures for PPIs/P-CABs increased by 9.0%, whereas those for ARBs, α-GIs, and statins decreased by 7.8%, 20.2%, and 5.8%, respectively. In Yamagata Prefecture, the PPI/P-CAB costs increased by 10.5%, whereas in Sakata, they decreased by 2.5%. A breakdown of these figures shows that in the prefecture, formulary drugs increased by 0.3% and non-formulary drugs by 13.2%, whereas in Sakata, formulary drugs increased by 32.8% and non-formulary drugs decreased by 14.1%. These results suggest that local formularies have a strong influence on prescription behavior.

The pharmaceutical expenditures for medical institution claims are listed in Table 4B. Costs decreased by 14.8% from 2018 to 2019 and by 5.1% from 2019 to 2020. Across all drug classes and regions, expenditures declined from 2018 to 2020, with Sakata showing a greater reduction than the rest of the prefecture.

**Fig. 1:**
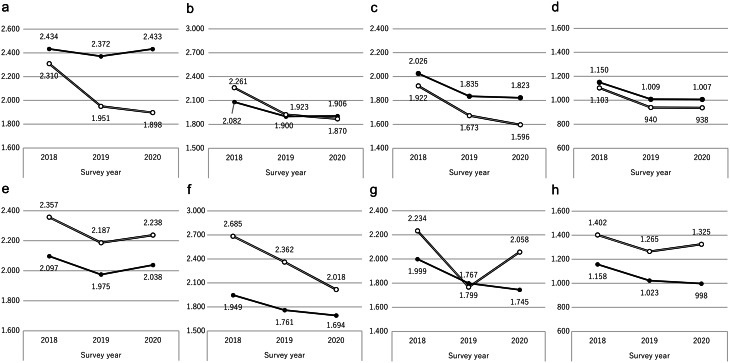
Average cost per claim (JPY). Drug costs were calculated based on the official drug prices for each survey year. Panels A–D show results from pharmacy claims, whereas panels E–H show results from medical institution claims. **A**,** E**: Unit cost comparison for proton pump inhibitor/potassium-competitive acid blocker drugs. ● Yamagata Prefecture, ○ Sakata. **B**,** F**: Unit cost comparison for angiotensin receptor blocker drugs. ● Yamagata Prefecture, ○ Sakata. **C**,** G**: Unit cost comparison for α-glucosidase inhibitor drugs. ● Yamagata Prefecture, ○ Sakata. **D**,** H**: Unit cost comparison for statins. ● Yamagata Prefecture, ○ Sakata

A comparison of unit costs per claim revealed that in pharmacy claims (Fig. 1, A–D), Sakata had lower unit costs than the prefecture for all drug classes by 2020. Sakata also showed a consistent year-on-year decline, whereas the prefecture experienced an increase in PPIs/P-CABs and ARBs from 2019 to 2020. For medical institution claims (Fig. 1, E–H), Sakata’s unit costs exceeded those of the prefecture for all drug classes in 2020. Notably, α-GIs and statins showed increasing unit costs in Sakata, diverging from the trends in the rest of the prefecture.

From 2018 to 2019, the rates of decline in unit costs for pharmacy claims were 2.6% for PPIs/P-CABs, 9.4% for ARBs, 8.8% for α-GIs, and 12.3% for statins in Yamagata Prefecture. In Sakata, the rates were 15.5%, 15.0%, 12.9%, and 14.8%, respectively. If Sakata’s rate of decline was applied to the entire prefecture, the estimated cost savings in 2019 would be ¥18,996,447 for PPIs/P-CABs, ¥13,860,132 for ARBs, ¥634,780 for α-GIs, and ¥2,363,138 for statins, totaling ¥35,854,497. A similar estimate for 2019–2020 yields an additional ¥15,034,247 savings (Table 5). Owing to drug price revisions in October 2019 and April 2020, direct comparisons of the pre- and post-revision costs were not valid. Therefore, this study used the annual change rates in unit costs, with the rest of Yamagata Prefecture serving as a control group.

**Table 5:** Estimated economic impact of local formulary results (cost, JPY)

Therapeutic class	Rate of change	2019			2020		
		Cost per claim	Total cost	Difference	Cost per claim	Total cost	Difference
PPI/P-CAB	Original	2,372	142,636,063	18,996,447	2,433	153,880,279	7,971,757
	Processed^a)^	2,056	123,639,616		2,307	145,908,522	
ARB	Original	1,900	204,722,573	13,860,132	1,906	207,443,233	6,402,824
	Processed^a)^	1,771	190,862,441		1,847	201,040,409	
α-GI	Original	1,835	16,316,740	634,780	1,823	15,263,968	604,596
	Processed^a)^	1,764	15,681,960		1,751	14,659,372	
Statin	Original	1,009	83,404,238	2,363,138	1,007	86,733,037	55,071
	Processed^a)^	980	81,041,100		1,006	86,677,966	
Total difference				35,854,497			15,034,247

^a)^Using data from Sakata

**Fig. 2:**
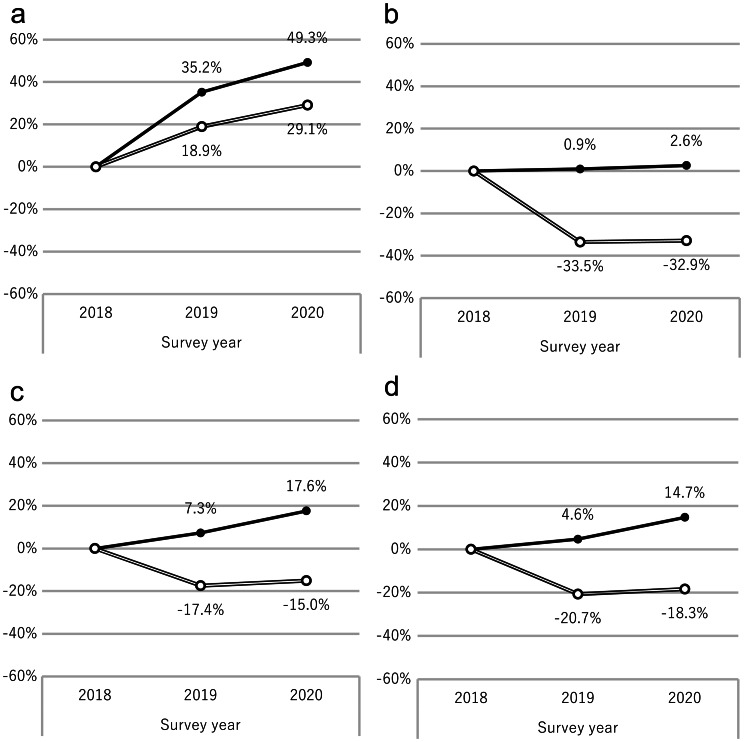
Prescription volumes of originator drugs without available generics. Prescription volumes were indexed to the 2018 baseline for each region, and annual changes are expressed as percentage ratios. **A**: TAKECAB^®^ (vonoprazan). ● Yamagata Prefecture, ○ Sakata. **B**: NEXIUM^®^ (esomeprazole). ● Yamagata Prefecture, ○ Sakata. **C**: AZILVA^®^ (azilsartan). ● Yamagata Prefecture, ○ Sakata. **D**: GLUBES^®^ (voglibose/mitiglinide combination). ● Yamagata Prefecture, ○ Sakata

The prescription volumes of originator drugs without available generics were compared annually, using 2018 as the baseline. In 2020, TAKECAB^®^ (vonoprazan, P-CAB) increased by 49.3% in the prefecture, but only 32.1% in Sakata. NEXIUM^®^ (esomeprazole, PPI), AZILVA^®^ (azilsartan, ARB), and GLUBES^®^ (voglibose/mitiglinide, α-GI) all showed increased usage in the prefecture, but decreased in Sakata by 32.9%, 15.0%, and 18.3%, respectively. These results indicate that the local formulary effectively suppresses the use of originator drugs (Fig. 2).

## Discussion

Sakata, the first region in Japan to implement a local formulary, established a regional medical cooperation corporation in April 2018 to implement a community-based integrated care system. One of the primary motivations for this initiative was the growing concern over the sustainability of healthcare and caregiving services owing to rapid depopulation, aging, and declining birth rates in the region. These challenges are common in many regional cities in Japan, and efforts to standardize and consolidate healthcare delivery systems to ensure quality care are expected to accelerate in the future.

In addition to workforce shortages, the increasing burden of social security expenditures poses a significant issue in Japan. According to the Ministry of Finance, the expansion of advanced medical care, aging populations, and growing use of high-cost pharmaceuticals have led to a continuous rise in healthcare spending [13]. Therefore, improving the efficiency of health care and curbing medical expenditure have become urgent priorities.

Local formularies, which are regionally tailored drug lists, promote the use of medications that are not only effective and safe but also economically advantageous. Consolidating therapeutically equivalent drugs contributes to more efficient pharmaceutical distribution and inventory management. Furthermore, when core hospitals and clinics within a region use the same active ingredients, seamless inter-institutional collaboration can be achieved, enhancing patient safety and reducing the workload of healthcare professionals. In June 2025, the Cabinet Office announced in its “Basic Policy on Economic and Fiscal Management and Reform 2025” that local formularies would be promoted not only to encourage the use of generic drugs but also to optimize healthcare expenditures [14].

This study examined the changes in prescription patterns resulting from the implementation of a local formulary using real-world data from the NDB. As of October 2020, the populations of the study regions were 935,183 in Yamagata Prefecture (excluding Sakata) and 133,513 in Sakata [15]. The numbers of claims per 1,000 people were 400 in the prefecture and 448 in Sakata, indicating no significant discrepancy in claims volume.

The analysis revealed that in pharmacy claims, Sakata showed higher adoption of formulary drugs across all drug classes compared with the rest of Yamagata Prefecture, confirming the promotion of formulary drug use. If this trend expands nationwide, it can potentially reduce pharmaceutical expenditures by several tens of billions of yen annually, demonstrating the significant contribution of local formularies to cost containment. Although this study focused on four drug classes, as of June 2025, Sakata has implemented formularies for 13 drug classes [16], suggesting further economic benefits.

In medical institution claims, Sakata showed increased formulary compliance, reduced unit costs in the ARB group, and higher compliance rates than the prefecture in the PPI/P-CAB group. However, for the other two drug classes, unit costs increased in Sakata, contrary to the trends in the rest of the prefecture, and no clear changes in prescription patterns or economic effects were observed. Possible reasons include limited drug inventories in hospitals and clinics compared with pharmacies; the need for formal procedures, such as approval by pharmaceutical committees to change adopted drugs; and the influence of stakeholder preferences. Additionally, the dissemination of information and educational activities by local pharmacists’ associations may not have reached medical institutions effectively.

Although there are reports of hospital formularies used to promote medication safety and appropriate use [17, 18], the extent to which economic factors should be considered remains unclear. In contrast, some core hospitals in Sakata actively promoted local formularies as part of their institutional policies, indicating variability in approaches across facilities. Therefore, a more detailed analysis of medical institution claims is necessary. However, owing to the risk of identifying individuals at the institutional level, such an analysis was not performed in this study.

This study also demonstrated that the implementation of a local formulary could suppress the prescription volume of originator drugs without the availability of generics. In Japan, incentives, such as the “Generic Drug Dispensing System Add-on” for pharmacies and the “Generic Name Prescription Add-on” and “Generic Drug Use System Add-on” for medical institutions, have been introduced to promote the use of generics. These incentives aim to replace originator drugs, whose patents have expired, with generics of the same composition. However, there are no direct restrictions or incentives regarding the prescription of originator drugs without generics, and originator drugs are often selected even when generics are available. This has been identified as one of the reasons why the expected cost-saving effects of generics are not fully realized. Local formularies represent the only initiative capable of suppressing the use of originator drugs without generics.

Moreover, although the economic benefits of switching to generics cannot be realized until patents for the originator drugs expire, the cost-saving effects of suppressing originator drug use are immediate. Therefore, the introduction of local formularies has played a significant role in maintaining the sustainability of Japan’s universal health insurance system.

In Japan’s drug pricing system, originator drugs eligible for the “New Drug Creation Premium” are generally exempt from price reductions during reimbursement revisions, whereas generics tend to experience larger price cuts. Consequently, in regions with high formulary drug usage rates, pharmaceutical expenditures are more likely to be reduced, and the economic effects of local formularies may be further amplified.

According to a report by the Japan Generic Medicines Association, as of December 2022, local formularies have been introduced in 15 regions across Japan [19]. As of July 2025, this number is estimated to have expanded to approximately 30 regions. There are also cases, such as in Takatsuki City, Osaka Prefecture, where local pharmacists’ associations and municipal governments have collaborated to initiate formulary implementation [20]. To date, no region has discontinued the use of a local formulary since its introduction, suggesting continued expansion. However, it is important that the implementation of local formularies is not driven solely by economic considerations but rather based on a multifaceted evaluation of pharmaceuticals and thorough regional discussions [21]. One example is the introduction of local formularies as part of disaster preparedness. By consolidating therapeutically equivalent drugs into formulary drugs, pharmacies can stock essential medications more easily, and mobile pharmacy vehicles dispatched during disasters can dispense medications more efficiently [22].

In Japan, there are eight active ingredients in ARBs and over 600 approved brand names, making it extremely difficult to dispense the same drug during a disaster. Moreover, under the current regulations, pharmacists are not permitted to substitute drugs without the physician’s consent, even during emergency prescriptions. Therefore, operating a common formulary at the regional level would enable a rapid response. Some regions have already begun to implement it, indicating its potential utility in future disaster preparedness [23].

To date, the macroeconomic effects of local formularies have not been empirically demonstrated, and their implementation remains limited in Japan. Consequently, the government has not yet introduced measures to promote adoption through reimbursement incentives. The introduction and maintenance of local formularies require considerable effort, and nationwide expansion will necessitate support from the government and insurers. In addition to evaluating the economic benefits, future studies should examine the therapeutic equivalence of patients who switch from non-formulary to formulary drugs. In hospital formularies, there are reports showing that switching to oral PPI/P-CAB drugs reduced medication costs without increasing event risk [24]. If similar findings are obtained for local formularies, healthcare professionals’ understanding and acceptance will further improve.

The limitations of this study include the fact that all target drug classes were used to treat chronic diseases and that prescription volumes may vary depending on seasonal factors. Therefore, extrapolating 1 month of data to the annual performance is difficult. Furthermore, because the NDB data source is not the most recent, the results cannot be directly applied to future projections, although they may be considered indicative of potential impacts.

Additionally, during the study period, some generic drugs in the PPI/P-CAB group were discontinued or subjected to shipment adjustments, which may have made the formulary drugs difficult to obtain. However, as supply issues were not limited to a specific region and this study compared prescription volumes and unit costs between the two regions, the impact on the results is considered limited. Although the coronavirus disease 2019 pandemic in 2020 may have affected healthcare utilization, the target drug classes were primarily used for chronic conditions, and the number of claims analyzed did not decrease, suggesting a minimal impact.

In this study, we examined the economic implications of local formularies in Japan using administrative claims data. However, it is important to note that a true pharmacoeconomic evaluation requires confirmation that therapeutic outcomes remain equivalent or superior following formulary implementation. Although Japan’s universal health insurance system differs from those in other countries, previous international reports have shown that restrictive formularies—such as those limiting access to antidepressants—can negatively impact patient outcomes and lead to increased hospitalization costs [25]. 

The fundamental role of local formularies should extend beyond short-term cost containment strategies, such as promoting the use of generic drugs. Instead, they should serve as a platform for supporting pharmacotherapy that prioritizes long-term economic sustainability and patient-centered care.

## Conclusions

The implementation of a local formulary led to an increased frequency of prescriptions for recommended drugs in outpatient settings within the target region. Simultaneously, the formulary suppressed prescriptions for originator drugs without available generic drugs, resulting in a synergistic effect that effectively reduced pharmaceutical expenditure. These findings indicate that local formularies can play a significant role in enhancing the sustainability of Japan’s national health insurance system.

## Data Availability

The data supporting the findings of this study are available from the corresponding author upon reasonable request.

## Abbreviations

NDB: National Database
PPIs: Proton pump inhibitors
P-CAB: Potassium-competitive acid blocker
ARBs: Angiotensin receptor blockers
